# Spiritual Care and Trauma REBOOT: An Interview Study of U.S. Course Graduates

**DOI:** 10.1007/s10943-025-02351-1

**Published:** 2025-06-21

**Authors:** Leanne K. Knobloch, Jenny L. Owens, Kirsten Pool

**Affiliations:** 1 Department of Communication, University of Illinois, 3001 Lincoln Hall, 702 South Wright Street, Urbana, IL 61801 USA; 2 REBOOT Recovery, Pleasant View, TN USA

**Keywords:** REBOOT Recovery, Spiritual care, Trauma, Trauma REBOOT

## Abstract

Spiritual care is in demand among individuals coping with trauma, but the barriers to seeking and providing spiritual care in traditional clinical settings suggest the need for community interventions. Trauma REBOOT is a 12-week, peer-led, Christian-based spiritual care course offered by trained volunteers in locations around the United States and online. We conducted the first study of the Trauma REBOOT program by interviewing 43 course graduates. Results revealed very positive perceptions of the course. Participants identified two reasons for attendance (seeking a program grounded in spirituality, looking for community), strengths of the program (structure of the curriculum, camaraderie among attendees), and avenues for improvement (preference for in-person format over remote format, more effective discussion leadership). They also described several effects of the course (more peace, transformed view of God, healthier boundaries in relationships, greater compassion for others, finding meaning in suffering). Based on these findings, we consider ways to enhance the delivery of spiritual care for trauma and refine the Trauma REBOOT program.

## Introduction

Worldwide estimates suggest that approximately 70% of adults will experience a traumatic event in their lifetime (Benjet et al., 2016; Kessler et al., 2017), often with major repercussions for their worldview and system of values. Trauma can damage people’s sense of self, disturb their faith in humanity, impede their trust in institutions, and shatter their feelings of safety (e.g., Park, 2022; Steinberg et al., 2022; Wortmann et al., 2011). By extension, people’s ability to heal from trauma can be deeply intertwined with their spiritual beliefs (e.g., Captari et al., 2022; Flint & Ronel, 2023; He & Petrakis, 2025). Religious and spiritual commitments correspond with better health in the wake of traumatic events (Hipolito et al., 2014; McIntosh et al., 2011; Shin et al., 2024; Upenieks & Ford-Robertson, 2022). Moreover, religious and spiritual difficulties can hinder trauma recovery (Captari et al., 2024). In short, spirituality can play an important role in healing from trauma (e.g., Captari et al., 2022; Flint & Ronel, 2023; He & Petrakis, 2025; Milner et al., 2020).

Spiritual care is a uniquely effective approach for helping individuals cope with trauma. *Spirituality* refers to the embodied and subjective experience of connection with the sacred and the transcendent (Captari et al., 2018); *spiritual care* refers to treatment services that address the sacred, the transcendent, and the spiritual dimension of existence (e.g., Daniel & Harris, 2024). Research demonstrates that spiritual care is helpful for reducing psychological distress, enhancing positive affect, and improving sleep (Knobloch et al., 2022; Reist Gibbel et al., 2019; Taylor et al., 2024). A meta-analysis of 97 outcome studies demonstrated that spiritual care is more efficacious on some metrics than standard psychotherapy treatment (Captari et al., 2018). Unfortunately, significant hurdles exist to providing spiritual care within traditional healthcare facilities (e.g., Currier et al., 2023; McGee et al., 2023).

REBOOT Recovery is a 501(c)(3) nonprofit organization with a mission to provide community-based spiritual care to trauma survivors. The organization offers a trio of courses funded by philanthropic donations and hosted by trained peer leaders in locations around the United States and online. REBOOT Recovery’s newest offering, the 12-week Trauma REBOOT course, was launched in 2020 to make spiritual care for trauma more accessible to people from all walks of life. Our study aims to conduct the first systematic investigation of the Trauma REBOOT program by soliciting the perceptions of course graduates in their own words.

### Spiritual Care Provided by Trauma REBOOT

Spiritual care for trauma can be difficult for people to access despite evidence of its efficacy (Captari et al., 2018). Many individuals express a desire to involve religious and/or spiritual issues in traditional mental healthcare but are reluctant to do so (Harris et al., 2016), often because of uncertainty about the provider’s openness to discussing spiritual beliefs, fear of judgment, and worry about mismatched values (Harris et al., 2016; He & Petrakis, 2025). Clinician barriers exist as well, particularly with respect to training and expertise (Currier et al., 2023; Jones et al., 2021). Only 8% of physical healthcare providers, 11% of psychologists and social workers, 21% of nurses, 26% of licensed professional counselors, and 31% of marriage and family therapists report having received formal training in spiritual care (McGee et al., 2023; Oxhandler & Parrish, 2018). Other practitioner impediments include the risk of offending patients, the limited time for care, and the tension between science and spirituality (Currier et al., 2023; Jones et al., 2021; McGee et al., 2023). Such hurdles imply the value of spiritual care for trauma offered outside of traditional clinical settings.

#### History of the Program

REBOOT Recovery (rebootrecovery.com), headquartered in Pleasant View, TN, has provided spiritual care to more than 30,000 trauma survivors and their loved ones since 2011. Its course offerings include Military REBOOT for service members and veterans dealing with military-related trauma, First Responder REBOOT for emergency personnel coping with critical incident trauma, and Trauma REBOOT for anyone grappling with the aftermath of trauma. All three programs are built around the core values of (a) conceptualizing trauma as an opportunity for growth, (b) learning from peers who have experienced trauma themselves, and (c) engaging trauma survivors’ loved ones in the healing process.

Trauma REBOOT was crafted during the COVID-19 pandemic in response to the urgent need for services. It is a 12-week course sequenced according to a manualized curriculum containing lesson plans, video content, discussion activities, and attendee workbooks (Owens & Owens, 2020). The curriculum is rooted in Christian spirituality and grounded in principles derived from social cognitive theory (Bandura, 1986, 2012), the transtheoretical model of health behavior change (Prochaska et al., 2008), and the occupational therapy practice framework (American Occupational Therapy Association, 2020). The curriculum defines trauma exposure as the experience of one or more deeply distressing and disturbing episodes, including physical abuse, emotional abuse, spiritual abuse, sexual abuse, neglect, violence, natural disaster, victimization, and death of a loved one (Owens & Owens, 2020). Individuals are eligible to attend if they self-identify as having experienced trauma.

#### Format of the Program

Face-to-face locations meet for 2 h per week in settings such as community centers, churches, and health clinics; remote locations meet for 1.5 h per week via an online conferencing platform. Participants are encouraged to attend with a trusted friend or loved one for support. The weekly topics are healing from trauma (Week 1), changing your default response to trauma (Week 2), understanding the spiritual wounds of trauma (Week 3), finding physical and emotional safety (Week 4), managing strong emotions (Week 5), grieving well (Week 6), overcoming guilt, shame, and regret (Week 7), restoring your identity (Week 8), sharing your story (Week 9), forgiving yourself and others (Week 10), finding stability (Week 11), and the graduation ceremony and moving forward (Week 12; Owens & Owens, 2020).

Individuals apply to REBOOT Headquarters for permission to lead a course in their local community or online. They complete an intensive online training program and receive personalized coaching to become certified as a peer leader. The training covers topics such as advertising the course, offering hospitality, presenting information, leading discussion, maintaining confidentiality, and safeguarding the emotional well-being of attendees. Some peer leadership teams raise funds and secure volunteers to offset the costs of providing course materials, food, and childcare.

In-person courses convene with a family-style fellowship meal and an opportunity for informal conversation. Afterwards, children and childcare staff move to a play area, and the peer leaders facilitate the lesson of the day in three modules. The kick-off module involves an opening prayer, a review of the previous session, and an ice-breaker activity. The lesson module can be delivered in one of two ways: (a) peer leaders can show a video taught by REBOOT headquarters staff with periodic pauses built in for discussion questions, or (b) peer leaders can teach the content themselves using a mix of lecture and discussion strategies. The wrap-up module includes an experiential homework challenge and an invitation to listen to a mid-week podcast located in the course app. Attendees follow along with a field guide workbook that contains content highlights, worksheets, reflection activities, scripture verses, and space to take notes.

#### Research Questions

Given the lack of prior data on the Trauma REBOOT program, we posed a trio of basic research questions to guide our investigation. First, we asked about course graduates’ reasons for attending the program (RQ1) to understand their motivations for enrolling. Second, we inquired about the most helpful and least helpful aspects of the course (RQ2) to gain insight into how attendees evaluate the program. Finally, we asked about any effects of the course (RQ3) to illuminate course graduates’ perceptions of outcomes.

## Methods

### Procedures

We first sought Institutional Review Board approval to conduct the study. After obtaining approval, we recruited participants by disseminating an announcement through an email listserv of course graduates maintained by staff at national headquarters. The announcement explained that REBOOT Recovery was collaborating with researchers from the University of Illinois to understand course graduates’ experience of the program. Interested recipients clicked on a link in the announcement to visit a webpage describing the study. After registering informed consent electronically, they completed a short demographic questionnaire and selected an interview slot.

A next step involved interviewer training. The third author, a doctoral candidate with expertise in the interpersonal effects of trauma but unaffiliated with REBOOT Recovery, received formal instruction about the program’s curriculum. Her preparation also included training in building rapport with interviewees, discussing challenging topics, and taking action in response to verbal and nonverbal expressions of emotional distress.

Participants utilized a computer, tablet, or phone to log into a secure online conferencing platform for the interview. To begin the session, the interviewer reviewed the informed consent agreement and asked for permission to audio record the conversation. Next, the interviewer administered a semi-structured interview protocol that included open-ended questions about various aspects of the Trauma REBOOT course (see Table 1); the interviewer probed if necessary for clarity. At the end of the session, the interviewer invited comments on any other topic not covered in the conversation. After the interview concluded, we emailed participants a set of national resources for dealing with trauma and a $50 e-gift card as a token of appreciation.

**Table 1:** Sample interview questions

What are your overall impressions of the Trauma REBOOT program?	
What are the reason(s) you decided to attend REBOOT?	
What aspects of the course were most helpful for you? Why?	
What aspects of the course were least helpful for you? Why?	
What would you change about REBOOT?	
What would have made your group’s leadership team better?	
How, if at all, did the course affect the way you see yourself?	
How, if at all, did the course affect your relationship with God?	
How, if at all, did the course affect your relationships with others?	
How, if at all, did the course affect the way you view your trauma experiences?	
What changes in your life, if any, resulted from REBOOT?	
How effective was REBOOT for helping you cope with trauma?	
What are the reasons that REBOOT was effective or not effective for helping you?	
What other services, if any, have you used to deal with trauma?	
How effective was REBOOT compared to any other approaches you have tried?	

### Participants

Participants were 43 trauma survivors (*n* = 14 men, *n* = 29 women) who graduated from the Trauma REBOOT program (see Table 2). Approximately 63% of individuals completed the course in person; the other 37% completed the course using an online conferencing platform. They graduated from the course in 2021 (5%), 2022 (23%), 2023 (51%), or 2024 (21%).

**Table 2:** Demographic characteristics of the sample

Demographic characteristic	*n*	%	*M*	*SD*
Biological sex				
Men	14	33		
Women	29	67		
Age (range = 23 to 89 years of age)			52.66	14.67
Race/ethnicity				
Non-Hispanic White	37	86		
Hispanic or Latino/a	4	10		
Asian	1	2		
Black or African American	1	2		
Educational achievement				
High school diploma or GED	15	35		
Associate degree	8	19		
Bachelor’s degree	13	30		
Advanced degree	7	16		
Relationship status				
Single	8	19		
Engaged to be married	1	2		
Married	17	40		
Separated	3	7		
Divorced	13	30		
Widowed	1	2		
Kinds of trauma				
Sexual abuse	12	28		
Childhood or parental abuse	9	21		
Domestic violence	4	9		
Abusive relationship	4	9		
Death of a loved one	3	7		
Combat trauma	3	7		
Occupational trauma	2	5		
Life-threatening illness	1	2		
Violent crime	1	2		
Declined to disclose	4	10		

*N* = 43 Trauma REBOOT graduates

Interviewees ranged from 23 to 89 years of age (*M* = 52.66, *SD* = 14.67, *Mdn* = 54). They identified as non-Hispanic White (86%), Hispanic or Latino/a (10%), Asian (2%), or Black or African American (2%). Their educational achievement was a high school diploma or GED (35%), an associate degree (19%), a bachelor’s degree (30%), or an advanced graduate degree (16%). They characterized their relationship status as single (19%), engaged to be married (2%), married (40%), separated (7%), divorced (30%), or widowed (2%). Approximately 72% were parents.

Most participants reported experiencing one trauma (63%), but others recounted multiple traumas (37%). Kinds of trauma included sexual abuse (28%), childhood or parental abuse (21%), domestic violence (9%), abusive relationship (9%), death of a loved one (7%), combat trauma (7%), occupational trauma (5%), life-threatening illness (2%), or violent crime (2%). Some interviewees chose not to disclose the kind of trauma they experienced (10%).

The interviews ranged from 28 to 65 min in length and averaged 47.19 min (*SD* = 10.85 min, *Mdn* = 49 min). We transcribed the interviews and double-checked them against the audio recordings for accuracy, which resulted in 699 single-spaced pages of text (range = 10 to 24 pages, *M* = 16.26, *SD* = 3.55) containing 276,141 words (range = 3387 to 11,097 words, *M* = 6421.88, *SD* = 1796.90). We removed identifying details from the transcripts before starting data analysis.

### Data Analysis

We selected *thematic analysis* as a comprehensive, robust, and flexible set of data analytic techniques (Braun & Clarke, 2021, 2022) particularly valuable for investigating issues of health and wellness (Braun & Clarke, 2014). Thematic analysis is designed to identify patterns of shared meaning in qualitative data (Braun & Clarke, 2021, 2022). From among the different versions of thematic analysis, we chose the *codebook approach* for two reasons. First, it melds precision with subjectivity by employing formal coding structures through an interpretivist lens (Braun & Clarke, 2022). Second, it is well-equipped for investigating applied research questions, managing large datasets, and organizing groups of coders working in tandem (Braun & Clarke, 2021, 2022).

Given the large amount of data, a team of eight coders collaborated on the thematic analysis over a 3-month period. The first and third authors led the process in conjunction with six advanced undergraduate research assistants majoring in health communication or health services. First, we trained the research assistants in the concepts, logic, and process of the codebook approach to thematic analysis. Then, the research assistants worked sequentially through the responses to each interview question to derive themes from the data. Following the six recursive phases of thematic analysis (Braun & Clarke, 2021), the coding team (a) gained familiarity with the data, (b) systematically coded the data, (c) generated provisional themes from the systematically coded data, (d) developed and refined the provisional themes in conjunction with the systematically coded data, (e) explicated and labeled the final themes, and (f) drafted the results. The research assistants coded independently to capitalize on their unique perspectives, but they met in person each week to share insights, interrogate claims, and resolve differences.

**Fig. 1:**
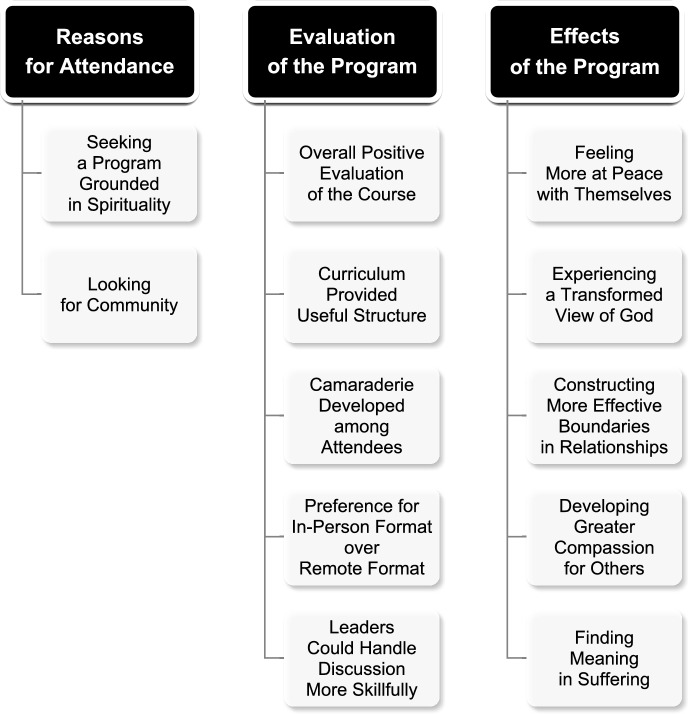
Results of the thematic analysis

We employed a variety of strategies to promote validity beyond adhering to best practices for thematic analysis (Braun & Clarke, 2021). Each week while data analysis was ongoing, the research assistants wrote analytic memos to enhance their reflexivity and examine their biases. Second, we labeled the provisional themes and the final themes using the vernacular participants employed in the interviews. We also sought to defend against a confirmatory bias by explicitly searching for opposing viewpoints in the data. Fourth, we utilized thick description and numerous direct quotations from participants in drafting our results. Fifth, we conducted member checks by requesting feedback from the 28% of the sample who agreed to review our preliminary results, and we integrated their comments into the final draft of the findings. We devote the following paragraphs to describing our results with direct quotations lightly edited for readability and identification numbers for privacy (see Fig. 1).

## Results

### Reasons for Attending the Trauma REBOOT Program (RQ1)

Participants identified two aspects of the Trauma REBOOT program that were appealing to them (see Fig. 1 and Table 3). First, interviewees reported *seeking a program grounded in spirituality*. “I was looking for a faith-based therapy program. I’ve had all the secular therapists I could handle,” said Participant 2. “It was very helpful for me to tackle the issues from a spiritual standpoint,” Participant 5 disclosed. Participant 19 commented that the program offered a different approach to trauma recovery:

Coping mechanisms, medications, nothing really healed me. I felt like my soul was torn. How do you heal your soul? REBOOT helped me heal as a whole person. Not just the mind, not just the brain, not just the physical side, but it literally helped me find myself on a soul level, a spiritual level.

Participant 26 prioritized the spiritual aspect of the program as well: “The main thing is that it’s faith-based. Without that, you can do all the other stuff, but it’s not going to matter.” Participant 31 said, “My faith is very important to me. I’ve gotten advice even from counselors and friends that just doesn’t really align with my beliefs. So having REBOOT be faith-based was really helpful for me.” The spiritual foundation of the program was a draw for some attendees.

**Table 3:** Reasons for attendance

1.	Seeking a Program Grounded in Spirituality
	“It had the religious component or the spiritual component to it, which is what I was looking for.”—*Participant 22*
	“There’s a lot of good things with having a spiritual aspect to the course. Any trauma therapist will tell you—whatever your religion or faith background is—to get in contact with a spiritual course.”—*Participant 9*
	“Of course, it is a faith-based program, which I think is imperative for really making a permanent change in one’s perspective of trauma.”—*Participant 25*
2.	Looking for Community
	“I was looking for something, trying to find that group, [REBOOT] was literally the only thing I could find. I think it’s really important to have some type of group support to collaborate and connect with.”—*Participant 21*
	“It was a good opportunity to connect with other people.”—*Participant 38*
	“Well, the main reason was education. And the secondary reason was you need community as well. Just hearing from other people.”—*Participant 34*

Other participants were *looking for community*. As Participant 9 stated, “I’ve been searching for a group because, as a trauma survivor, you always feel like you’re a step outside of normal social settings. You just don’t feel like you fit in. So that’s what I was looking for.” Participant 25 echoed the need to build social ties: “My biggest desire in the course was to make connections with those who could understand, because that is a huge need for someone who is trying to move forward and recover.” Similarly, Participant 14 enrolled in Trauma REBOOT because “there was group support. That’s huge for me because I want others to be able to relate to me, and I want to be able to relate to them, and to provide that support for each other.” Participant 42 sought community as well, “I missed that connection of belonging, of really getting connection. And I’ve noticed that’s a consistent need throughout my life, too.” The small group setting of the Trauma REBOOT program was attractive to participants seeking fellowship.

### Evaluation of the Trauma REBOOT Program (RQ2)

To gain insight into how course graduates evaluated the Trauma REBOOT program, participants responded to a general question about their experience of the course, and then they answered specific questions about the most helpful and least helpful aspects of the program (see Table 1). Findings revealed an overwhelmingly positive response to the course, plus two areas of strength and two targets for improvement (see Table 4).

**Table 4:** Evaluation of the program

1.	Overall Positive Evaluation of the Course
	“Therapeutic.”—*Participant 29*
	“I can’t say enough good things about a program that literally changed my life.”—*Participant 19*
	“It’s helped me with my relationship, keeping a job, just dealing with just the ups and downs of life.”—*Participant 1*
	“It allowed me to heal a lot more.”—*Participant 23*
	“Very informative, completely supportive.”—*Participant 2*
	“Well designed, impactful, and meaningful.”—*Participant 37*
	“We looked forward to every session and walked away really with so much. So excited to see the growth in all the people and the healing that was occurring.”—*Participant 41*
	“I’ve been very impressed with it.”—*Participant 28*
2.	Curriculum Provided Useful Structure
	“REBOOT as a curriculum has got plenty of good things in there to help you identify what’s going on and then help you cope with them.”—*Participant 36*
	“The content of the course was beyond helpful.”—*Participant 27*
	“Going through the content and having that guided workbook was really good because it’s easy to put something off. When you have it in a workbook form and you have that guidance, like, this is what you’re going to do.”—*Participant 24*
3.	Camaraderie Developed among Attendees
	“We did it together as a group. You share and get to know everybody else’s trauma in a very personal way. Things they have never said to another living person. And so you’re processing that together, which is so powerful.”—*Participant 15*
	“Finding my voice with others, and there’s a support group in the community that I have never felt before.”—*Participant 19*
	“I liked the idea that it was in a group. There’s so much support. You learn from others, you help others, and they help you.”—*Participant 30*
4.	Preference for the In-Person Format over the Remote Format
	“The virtual component was tough. I think we lived very far away from each other, so that’s tricky.”—*Participant 12*
	“The only thing that comes to mind is maybe not virtual but just in person.”—*Participant 32*
	“We had women who refused to be on camera, and no matter how hard [the leaders] tried to get the women to be on camera, they weren’t able to convince them to do so.”—*Participant 8*
5.	Leaders Could Handle Discussion More Skillfully
	“The co-leader, she was incredible. She was compassionate, she was understanding, she was willing to take the time. But the main leader, I felt like, was in a hurry.”—*Participant 40*
	“A leader in our group talked a little too much, and she focused on her experience and not really the people in the class. Someone talked to her about that, but then it got kind of weird and awkward, because I think she felt like she couldn’t talk at all.”—*Participant 16*
	“I felt like there were some people who wanted to be leaders instead of participants, and they were allowed to talk over people. And it can be hard as a leader to say, ‘Hey, listen. It’s not really your spot.’”—*Participant 43*

Strong praise was offered for the Trauma REBOOT program. Interviewees labeled it “incredibly effective” (Participant 35), “wonderful” (Participant 6), “really eye-opening” (Participant 18), “awesome” (Participant 20), “very supportive and very helpful” (Participant 8), and “a hundred percent positive” (Participant 39). Participant 12 complimented several aspects of the course: “It was super helpful. I learned a lot. It was a great launching point into a healing journey, and I was really impressed with the people involved.” Other participants explained their growth from the program. For example, Participant 4 asserted, “It has transformed my life. The way I do things, interact with my family, speak to anybody—transformational.” Participant 22 commented, “I’m definitely living a better life using the tools I acquired from Trauma REBOOT. Better decisions, more confident, even down to my eating habits, exercising, so many things. It’s affected my whole life.” These statements and many others showcased a widespread favorable impression of the course.

A specific strength was that the *curriculum provided useful structure*. Participant 30 remarked, “The different weeks and the different topics were just very helpful.” For Participant 17, “It was the structure and it was the accountability.” Participant 15 made a similar point: “This is very structured and keeps you on topic. That’s what I like about it.” Other participants described their preference for a course format over a more open-ended clinical approach. For example, Participant 16 noted:

I had tried a lot of therapists, and all they were saying was “You’re fine. You’re not wrong or crazy,” or whatever. But I needed a more structured class to delve into what the traumas did to me and how to overcome them. And so this was really helpful because I wasn’t just getting the same answer all the time. I was actually getting some help on how to overcome my traumas with this class.

Participant 21 favored the linear organization of Trauma REBOOT over the more nebulous progression of therapy as well:

I would say the educational process. There were stages, they gave us a book, and it was a step-by-step process. You actually had a goal in mind. I mean, you can go to therapy and it’s an all-generic goal. “I don’t want to feel this way anymore.” “Okay, great.” There’s no real timeframe. But [REBOOT] provides a sense of accomplishment and actual steps you can see yourself taking. It actually has a structure to it, and it has steps to build on.

Participants appreciated how the course content and design were geared toward building momentum, tracking progress, and maintaining accountability.

Another valuable aspect of the course was that *camaraderie developed among attendees*. Participant 25 highlighted how much those bonds meant to him: “It was lifesaving for me to be able to find someone and have common ground. And then we have transparency and genuineness that we don’t ever have with anyone else.” Similarly, Participant 33 remarked, “I would say the togetherness of the group. Talking, listening to people talk about their trauma, you being able to talk about your trauma with them, and just being understood.” Participant 41 echoed the point: “I was really impressed with how it brought everybody together.” Participant 34 appreciated “having a community that comes together, and you know, is willing to step forward.” Such statements applauded the tight-knit connection that emerged among Trauma REBOOT graduates.

With respect to the least helpful aspects of the program, participants expressed a *preference for the in-person format over the remote format*. As Participant 11 acknowledged, “People in trauma tend to isolate, and having that physical engagement is part of the healing process. On Zoom it’s a lot easier to just drop out and not be as engaged.” Participant 1 remarked, “The virtual part is hard because you’re missing a human element. There’s something about being in the presence of a human being, and technology can never replace that.” Participant 14 agreed: “I would prefer it to be in person. These days, everything’s online, and it takes away that communal healing aspect.” Participant 3 experienced both formats: “I did it via Zoom and via in-person, and I much preferred the in-person because you got to really connect with others.” Although participants endorsed in-person sessions most frequently, opposing opinions also were apparent in the data. For example, Participant 27 said, “Virtual is everything, and it still is for me. I don’t know that I’d want to be in an in-person course.” Only 37% of our sample completed Trauma REBOOT using an online format, but a notable subset of those participants favored in-person meetings.

A second critique was that *leaders could handle discussion more skillfully*. Although several participants praised the leaders of their course (Participant 6: “My leaders were phenomenal.” Participant 10: “My leader was very well versed.” Participant 38: “We had great leadership.”), others mentioned ways their leaders could facilitate conversations more effectively. For example, Participant 13 recounted that “one of the leaders had a tendency to repeat what you said in her perspective, not yours. It didn’t feel authentic.” Participant 11 stated, “They spent most of our time talking, and so a lot of our participants didn’t really have that open time.” Participant 24 had the opposite experience: “The facilitator was a little bit like a drill sergeant. Not wanting anybody to share too long.” Participant 37 called for more organization: “The leadership team should’ve all known what they were sharing beforehand. It was more off-the-cuff.” Such comments, viewed as a set, implied that course leaders performed somewhat unevenly when facilitating discussion.

### Effects of the Trauma REBOOT Program (RQ3)

The interview contained a series of questions about how, if at all, the course affected the way participants saw themselves, their relationship with God and others, and their view of trauma (see Table 1). A final question asked about any other changes to their life as a result of the program. Five effects of the course were apparent (see Table 5).

**Table 5:** Effects of the program

1.	Feeling More at Peace with Themselves
	“I need to go easier on myself and forgive myself more. I really did do the best that I could in the situations I was given.”—*Participant 14*
	“I’m my biggest cheerleader now. I wake up every morning and I look in the mirror and I remind myself that I am important, that I am loved, that I am valued, that I’m all the things I tell everybody else.”—*Participant 4*
	“Knowing I did the best that I possibly could at that time. Could’ve, would’ve, should’ve, you know. It really releases the weight.”—*Participant 20*
2.	Experiencing a Transformed View of God
	“It helped for me to say, ‘Okay, God, let me see this [situation] through your eyes. Help me to feel this through your eyes, and once it’s felt, what is the next step?’ And to bring in that joy.”*—Participant 13*
	“Just the encouragement of how faithful God is. He can turn these crazy terrible situations for our good, and he can use all the pain. He doesn’t waste it.”—*Participant 31*
	“It brought me comfort knowing that God is with me the whole time, and I’m still here because of God. Even the other participants in the program, we were all placed together for a reason. It was just beautiful evidence of God being there with us.”—*Participant 14*
3.	Constructing More Effective Boundaries in Relationships
	“It’s allowed me to open up to my family who participated in my abuse. There are family members who I no longer speak to—I had to cut them out of my life because of boundary reasons. But one [sibling] and I discuss it quite often.”—*Participant 2*
	The program “helped me make some healthy boundaries, and say, ‘Okay, I can forgive from a distance. I don’t have to be in the same place as that person.’”—*Participant 22*
	Trauma REBOOT “allowed me to set better boundaries, which I’ve always heard about, but I’m actually able to do that now.”—*Participant 17*
4.	Developing Greater Compassion for Others
	“I think going through the course in general just builds more empathy for others.”—*Participant 26*
	“It helped my relationships. I’m actively trying to build skills so that I can be a better friend and more helpful to other people.”—*Participant 31*
	“Being able to interact with people knowing that there could be traumatic experiences they’ve dealt with. It lets me be more kind to them.”—*Participant 37*
5.	Finding Meaning in Suffering
	“The way I see it is, I don’t choose my trauma, but it’s something I had to go through in order to come out as who I am today.”—*Participant 18*
	“I look at [my trauma] differently, as in, it’s given me some very beautiful things in life and not just awful anxiety. So I’m thankful that it has made me into the person I am.”—*Participant 9*
	The program “helped me realize that my hurt has been able to help others heal.”—*Participant 35*

Interviewees reported *feeling more at peace with themselves* because of the Trauma REBOOT program. “It’s been so freeing,” Participant 8 remarked. “I just filed for divorce and now I’m like, ‘Okay, time to clean up my house. Let’s go.’ And it’s amazing how just releasing one more step of the trauma, it’s been so freeing.” According to Participant 9, the Trauma REBOOT program “helped me look at myself in a more positive light—that I’ve got something to offer.” Participant 39 also depicted a weight lifted: “The confidence, I would say, and my peace of mind. It’s helped me be more relaxed. I didn’t realize I was holding a lot in. Now there’s a little more peace and calmness.” Participant 35 gained more self-acceptance: “I’m definitely more gracious with myself, where I wasn’t in the past. I was very critical. I would talk down about myself. I wouldn’t do that to other people, but I was very negative all the time about myself.” Such comments illustrate how the course helped individuals gain confidence and unload shame.

Participants also mentioned *experiencing a transformed view of God*. Participant 15 observed, “I saw a loving father in [my trauma] that I needed. And the relief of relying on something outside of myself.” Participant 18 said Trauma REBOOT furnished insight into the fidelity of God, “It helped me see what God’s brought me through, and how there’s the same pattern over and over of his faithfulness. He’s shown his faithfulness.” Participant 1 described a revised perspective on God’s role in suffering:

I remember telling God, “Why didn’t you kill me? One bullet to the head would’ve just taken care of it. Look at me now. I’m out of control.” REBOOT helped me manage that. Now I see God as a healer, as a father who heals the wounds of his children.

Participant 19 also experienced a shift in his image of God: “My question is, ‘Where was God when all this was going on? From the house fire, to the shootout, to the things that happened in my childhood, ‘Why?’ is the big question. REBOOT, toward the end, you understand why.” These comments, and others like them, showcased people’s shift from anger to openness toward God.

Participants delineated how Trauma REBOOT helped them with *constructing more effective boundaries* in their relationships. Participant 12 disclosed that the course “helped me to have healthier relationships with people in my life. My relationships are more reciprocal and have better boundaries.” Participant 16 remarked, “I realized that I can’t be responsible for other people’s actions, and I’ve got to live my own life.” For some course graduates, the process of erecting stronger boundaries damaged some interpersonal ties but enhanced others. Participant 4 noted: “Healthy relationships is a big one for me. The sad thing is, I had to walk away from family because they were holding it against me, and I couldn’t let them play those games anymore.” Similarly, Participant 5 learned that “setting boundaries with people is okay. Some relationships have ended or have been cut drastically, but other ones have improved a lot.” Overall, course graduates felt more satisfied with their social lives despite having to eliminate some relationships from their networks.

A second effect of the course on people’s relationships involved *developing greater compassion for others*. Participant 17 characterized the change in this way: “It made me more empathetic with other people, and it also improved my vision so I can see people who are hurting and speak to their pain.” Participant 24 identified a similar evolution in how she interacts with others: “I feel like there’s a peace in me, so then I can respond to other people more lovingly instead of, like, panic.” Participant 20 mentioned having more insight into the perspectives of others: “It made me way more understanding of people going through trauma and what things might affect them.” Participant 7 identified spillover from the course to family members: “It increased my compassion for my children because they’ve been through trauma also. Maybe it increased my patience.” In other words, the Trauma REBOOT course helped to spark more empathy for others.

Interviewees also felt that Trauma REBOOT aided them in *finding meaning in suffering*. More specifically, participants related how they were using their experiences as an opportunity to serve others. For example, Participant 38 said that the course “redefined [my trauma]. It sucks that it happened, but it can turn into a really good thing—a way to help.” Participant 28 observed, “Even though there was pain and all the bad stuff, God connected me with Trauma REBOOT with a purpose to help. I feel like I’m supposed to help others with trauma in some way.” Participant 22 explained looking towards the future with acceptance: “I’ve moved forward, and I know that God is using [my experiences] for good, not for shame, or condemnation, or blame.” Participant 3 said simply, “There’s a purpose behind it. There’s a purpose behind that pain.” In other words, the course encouraged participants to think about their trauma in a new light.

## Discussion

Trauma has well-established effects on physical and mental health, but it also has ramifications for spiritual health (e.g., Captari et al., 2022; Milner et al., 2020). The close connection between spirituality and healing from trauma accentuates the importance of supplying high-quality, evidence-based, easily-accessible spiritual care for trauma. In addition, the impediments to offering spiritual care in clinical settings highlight the need for community-based programs. Trauma REBOOT was created in 2020 in response to those gaps. Our study provided a first examination of Trauma REBOOT by soliciting course graduates’ perceptions of the program in their own words. We devote the following paragraphs to synthesizing our results, delineating implications for practice, and identifying limitations and directions for future research.

### Reasons for Attendance

A first step in understanding the Trauma REBOOT program was to examine why attendees chose to enroll. When asked about the reasons they attended the course, interviewees mentioned being attracted to both the spiritual and the community dimensions of the program (RQ1). Obviously, individuals with deeper faith commitments may be more inclined to seek spiritual care (see Harris et al., 2016), and our qualitative findings cohere with quantitative results showing that graduates of the Military REBOOT program report more intrinsic religiosity than a nationally representative sample of U.S. veterans (Knobloch et al., 2022). Spiritual care is not a good fit for everyone (Harris et al., 2016), so insight into individuals’ motivation for attending the program is valuable for illuminating why they seek spiritual care.

### Evaluation of the Program

Participants’ overall response to the Trauma REBOOT course was very complimentary. Virtually all interviewees depicted satisfactory experiences of the program: “magic” (Participant 36), “much needed” (Participant 34), “a real support” (Participant 42), “saved and changed my life” (Participant 27). They identified two especially helpful aspects of the program: (a) a structured curriculum that provided useful content, and (b) a sense of camaraderie that developed among attendees (RQ2). Notably, both of those components diverge from orthodox approaches to trauma treatment (e.g., Guideline Development Panel, 2019): The structured curriculum is more regulated than the open-ended atmosphere of traditional psychotherapy, and the camaraderie between attendees requires a group format instead of a standard one-on-one clinical setting. These findings make sense given that many Trauma REBOOT participants anecdotally report enrolling in the course after exhausting other treatment options, but at the same time, our data suggest that spiritual care programs may do well to capitalize on manualized content and peer fellowship.

Areas of improvement involved both the format and leadership of the course (RQ2). A consistent theme among the 37% of the interviewees who completed the course via an online conferencing platform was the desire to participate in a face-to-face venue. Some course graduates also wished their peer leaders were more proficient at facilitating group discussions. On a micro level, this feedback is valuable for helping the REBOOT Recovery staff improve course delivery and train peer leaders more effectively. On a macro level, such findings highlight a pair of inherent tensions facing organizations offering spiritual care programs. One is the trade-off between accessibility and engagement: Remote channels maximize convenience for attendees, but they sacrifice the physical presence helpful for building community (e.g., Popper-Giveon & Keshet, 2024). Another is the trade-off between peer leadership and clinical administration: Peers have built-in solidarity with attendees due to their shared experiences, but they lack the professional competencies of clinicians (e.g., Jones et al., 2021; van de Ven, 2020). Our data imply that both tensions require strategic planning by spiritual care teams to ensure optimal delivery.

### Effects of the Course

Interviewees referenced several ways the course affected them. They reported feeling more at peace with themselves, experiencing a transformed view of God, constructing healthier boundaries in relationships, developing more compassion for others, and making sense of their trauma (RQ3). These themes cohere with prior interviews conducted with military personnel completing the Military REBOOT course (Knobloch et al., 2021) and emergency personnel completing the First Responder REBOOT course (Knobloch & Owens, 2023b). Perhaps more importantly, our open-ended and retrospective findings are consistent with the closed-ended and prospective reports of graduates of the other two courses (Knobloch & Owens, 2023a; Knobloch et al., 2022). Initial evidence is promising that attendees receive the Trauma REBOOT course at a level comparable to the more established offerings in the REBOOT Recovery portfolio.

A particularly intriguing result was how attendees’ view of God changed over the 12-week program. They described a shift from perceiving God as withdrawn and/or punitive to engaged and/or compassionate. Not only do these findings mirror the transformed view of God that Flint and Ronel (2023) observed in qualitative interviews with trauma survivors who credited recovery to spiritual growth, but they cohere with Exline et al.’s (2017) quantitative results demonstrating more growth after trauma among individuals who see God as having a benevolent role in suffering. They also triangulate Kim and Currier’s (2025) quantitative findings that those who believe God should have prevented their suffering may be most vulnerable to distress. All three research teams speculate about clinical strategies for helping trauma survivors reappraise their view of God (Exline et al., 2017; Flint & Ronel, 2023; Kim & Currier, 2025); our data imply that Trauma REBOOT may have value as such a strategy.

Another notable finding was that Trauma REBOOT graduates reported acquiring both more independence from loved ones and more intimacy with loved ones as effects of the program. One on hand, they learned how to establish better boundaries with others as a way of protecting themselves and maintaining more equitable relationships. At the same time, they gained a deeper appreciation for other people’s struggles and more compassion for those in their social circles. A striking aspect of these testimonies is how they echo the fundamental pull between autonomy and connection that lies at the heart of interpersonal relationships (e.g., Baxter & Montgomery, 1996; Feeney & Fitzgerald, 2022). In fact, theories of human relating contend that people’s ability to balance their competing desires for autonomy and connection is an important determinant of relationship success (Baxter & Montgomery, 1996). Trauma REBOOT may help attendees navigate a key obstacle to maintaining satisfying relationship ties.

### Limitations and Directions for Future Research

Several limitations of our investigation set an agenda for additional work. For example, our recruitment procedures targeted those who graduated from the 12-week course. A next step is to solicit the vantagepoints of individuals who considered Trauma REBOOT but did not enroll, attend, and/or complete the program to shed light on obstacles to uptake (e.g., Samuels et al., 2021). Second, we employed voluntary response sampling methods to populate our study. More sophisticated population sampling techniques are important for subsequent projects to mitigate the possibility of self-selection biases in people’s evaluation of Trauma REBOOT (e.g., Stone et al., 2024). Third, we focused on trauma survivors in this first examination of the course, but the program welcomes the attendance of loved ones to support the healing process. A follow-up study is needed to consider whether spiritual care programs such as Trauma REBOOT are effective for alleviating secondary distress among friends and family members of trauma survivors (e.g., Knobloch & Owens, 2023a; Knobloch et al., 2019).

Other limitations involve the type of data we collected. First, we interviewed course graduates at only one point in time. With cross-sectional evidence now implying a favorable response to the program, future research could consider a sequenced interview design to clarify whether attendees’ perceptions of the course change over time (e.g., Anandarajah et al., 2021). Second, although our qualitative interview approach was helpful for illuminating course graduates’ open-ended perceptions of Trauma REBOOT, quantitative survey measures are valuable for benchmarking any potential effects (e.g., Knobloch & Owens, 2023a; Knobloch et al., 2022).

Other directions for future research could build upon this study to expand the scope of knowledge about spiritual care for trauma. For example, qualitative and quantitative work examining REBOOT Recovery courses alongside other spiritual care interventions such as religiously integrated cognitive behavior therapy (Pearce et al., 2015), ACTing Spiritually (Ford et al., 2024), Winding Road (Reist Gibbel et al., 2019), Building Spiritual Strength (Harris et al., 2018), and dignity therapy (Samuels et al., 2021) could drill down into the most effective aspects of each approach. Such a study also could shed light on the core mechanisms responsible for fostering improvement. Is the pivotal force an ability to make meaning (e.g., Park, 2022), or a transformed view of God (e.g., Flint & Ronel, 2023), or a healthier balance between autonomy and connection in relationships (e.g., Baxter & Montgomery, 1996)? Understanding the key driver at work could pave the way for even more efficacious spiritual care interventions (e.g., Captari et al., 2022). In addition, mixed-method research delving into the gateways to participating in spiritual care programs could illuminate ways to facilitate access. Such work could inform public policy to ensure that spiritual care is available where and when individuals need it (e.g., Hall & Powell, 2021).
